# Posttraumatic Stress Disorder and Co-occurring Addictions: A Narrative review focusing on approach and therapeutic challenges

**DOI:** 10.1192/j.eurpsy.2025.988

**Published:** 2025-08-26

**Authors:** C. A. Romano, C. P. Osório, J. M. Paupério

**Affiliations:** 1 Psychiatry and Mental Health, Tâmega e Sousa Hospital Centre; 2 Psychiatry and Mental Health, São João, Oporto, Portugal

## Abstract

**Introduction:**

Posttraumatic Stress Disorder (PTSD) and substance use disorders (SUD) frequently co-occur, and their combination presents significant challenges in diagnosis and treatment due to the complex interaction between trauma-related symptoms and substance misuse behaviors.

**Objectives:**

This review aims to explore the pharmacological and psychosocial interventions conducted in comorbid PTSD and SUDs. It specifically focuses on which interventions are effective in addressing substance use as opposed to post-traumatic symptoms in patients with these concurrent conditions.

**Methods:**

Narrative literature review by performing a search on MedLine for English-written articles. The following search terms were used: ((“posttraumatic stress disorder”) AND (“substance use disorder” OR “alcohol use” OR “cannabis use” OR “cocaine use” OR “opioids use”) AND ((“treatment”) OR (“adherence”))).

**Results:**

Studies show that 46,4% of individuals meeting the criteria for PTSD also meet the criteria for an SUD, with an additional correlation between PTSD symptomatology and an increased substance use frequency and associated heightened risk of other mental health concerns, suicidality, mortality, and functional impairment. Within treatment-seeking samples, patients with PTSD are approximately 14 times more likely to meet the criteria for a SUD than patients without PTSD, with alcohol and cannabis as the most frequent substances involved. In clinical settings, management involves employing treatment strategies effective for each condition independently. The literature is not uniform regarding the impact of substance consumption in the treatment of PTSD, although it suggests a greater impact of alcohol when compared to other substances, in which studies are relatively uniform in highlighting a minor effect. Despite this minor effect, literature advances neurocognitive impairments and higher psychosocial stressors (legal issues, financial challenges, unemployment, housing instability, and a decline in social support) as potential causes leading to poorer outcomes. Despite this, there seems to be consensus on the fact that SUD was associated with the highest rate of discontinuation from both psychotherapy and medication treatment for PTSD.

**Conclusions:**

Concurrent PTSD and SUD present complex clinical challenges requiring integrated treatment approaches addressing both trauma-related symptoms and substance use behaviors. This narrative review underscores the importance of comprehensive assessment, evidence-based interventions, and multidisciplinary collaboration in facilitating recovery and improving functional outcomes.

**Disclosure of Interest:**

None Declared

